# Signaling of Human Frizzled Receptors to the Mating Pathway in Yeast

**DOI:** 10.1371/journal.pone.0000954

**Published:** 2007-09-26

**Authors:** Dietmar Dirnberger, Klaus Seuwen

**Affiliations:** Novartis Institutes for Biomedical Research, Basel, Switzerland; Ecole Normale Superieure, France

## Abstract

Frizzled receptors have seven membrane-spanning helices and are considered as atypical G protein-coupled receptors (GPCRs). The mating response of the yeast *Saccharomyces cerevisiae* is mediated by a GPCR signaling system and this model organism has been used extensively in the past to study mammalian GPCR function. We show here that human Frizzled receptors (Fz1 and Fz2) can be properly targeted to the yeast plasma membrane, and that they stimulate the yeast mating pathway in the absence of added Wnt ligands, as evidenced by cell cycle arrest in G1 and reporter gene expression dependent on the mating pathway-activated *FUS1* gene. Introducing intracellular portions of Frizzled receptors into the Ste2p backbone resulted in the generation of constitutively active receptor chimeras that retained mating factor responsiveness. Introducing intracellular portions of Ste2p into the Frizzled receptor backbone was found to strongly enhance mating pathway activation as compared to the native Frizzleds, likely by facilitating interaction with the yeast Gα protein Gpa1p. Furthermore, we show reversibility of the highly penetrant G1-phase arrests exerted by the receptor chimeras by deletion of the mating pathway effector *FAR1*. Our data demonstrate that Frizzled receptors can functionally replace mating factor receptors in yeast and offer an experimental system to study modulators of Frizzled receptors.

## Introduction

The mammalian Wnt proteins are secreted lipid-modified signaling molecules which control aspects of embryonic development. In the adult, Wnts have a role in diseases such as cancer, osteoporosis, and psychiatric disorders (reviewed in [1], [2]). Wnts function via receptor-mediated signaling pathways including members of the Frizzled (Fz) gene family.

Frizzled receptors share the basic structural organization of G protein-coupled receptors (GPCRs) with seven transmembrane-spanning domains, and exhibit a complex N-glycosylated exofacial N-terminal region. With regard to signal transduction, the majority of literature has dealt with the so called canonical signaling pathway which is quite divergent from classical GPCR signaling. In the canonical pathway, the phosphoprotein Dishevelled (Dv1) inhibits glycogen synthase kinase 3-β (GSK3β) leading to stabilization and nuclear translocation of the β-catenin protein and transcription of specific response genes (for a recent review see [3]).

Interestingly, there is also evidence that Frizzled receptors can exert functions independently of β-catenin. These phenomena were called ‘noncanonical’ Wnt-Fz signaling and common features with classical GPCR signal transduction were described (reviewed in [4], [5]). This includes sensitivity of Wnt-stimulated calcium flux and other signaling events to pertussis toxin, a specific inhibitor of G proteins of the G_i_-class. Furthermore, Wnt-11-stimulated Rho GTPase activity was shown to depend on Gβγ signaling. Frizzled receptors were also described to couple to the visual G protein transducin (G_t_). Dishevelled was found to recruit β-arrestin, a known modulator of GPCR function, to regulate Wnt5A/Fz4 signaling in human cultured cells [6]. Some reports also implicated heterotrimeric G proteins in canonical signaling stimulated by rat Frizzled-1 (Rfz1) [7], [8]. Strong evidence in this direction was also reported in *Drosophila*, where genetic experiments revealed that Gα_o_ was required to transduce both canonical and non-canonical signals [9]. However, a direct role of Frizzled receptors as GDP-GTP exchange factors for Gα proteins has not yet been demonstrated.

**Figure 1:**
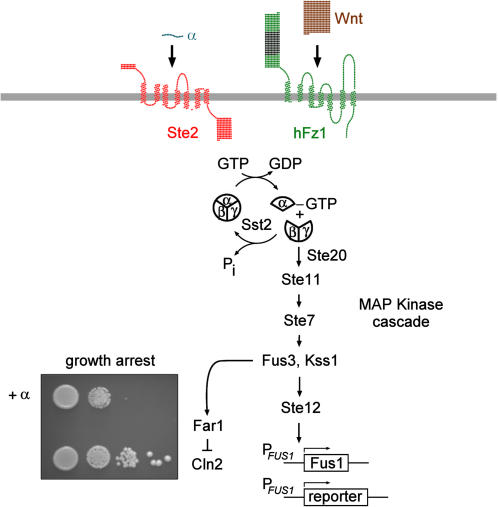
Schematic representation of the GPCR-controlled mating pathway in *S. cerevisiae*. Shown is the situation for mating type a haploid yeast cells. Cells normally express the α-factor receptor Ste2p – Frizzled receptors are introduced by ectopic expression. Ste2p activation through its ligand α-factor leads to GDP-GTP exchange in the Gα protein Gpa1p, and dissociation of the heterotrimeric G protein. The resulting free βγ subunits trigger activation of a MAP kinase cascade leading to the transcription of pheromone-responsive genes such as *FUS1*, as well as to G1 growth arrest via inhibition of the cyclin-dependent kinase inhibitor *FAR1* gene product. A mating pathway activation growth assay using wildtype yeast cells (MH272-1da) is shown bottom left. The strain was spotted in serial dilutions (∼1E4, 1E3, 1E2, 1E1 cells in duplicate) onto complete minimal medium, and treated with 1 µg α-factor spotted directly onto the yeast patches as indicated.

Key principles of GPCR signaling have been unraveled using yeast as a model system. This includes the genetic demonstration of signaling through Gβγ proteins, as well as the identification of the first member of the regulator-of-G protein-signaling (RGS) class of Gα GTPase activators, termed Sst2p [10]. In yeast, GPCRs control the mating pathway that is used for sexual reproduction (reviewed in [11]). When haploid cells of mating type a and α meet, they extend a projection toward the other (“shmoo”), stop their cell cycle, start transcription of mating genes, and finally fuse to form a diploid cell. The mating process involves specific signaling proteins such as secreted pheromones (**a** and α-factor), GPCR-class pheromone receptors (Ste2p and Ste3p), a heterotrimeric G protein (Gαβγ), a Gβγ - activated MAP kinase cascade leading to expression of pheromone-responsive genes, and growth arrest via CDK-inhibition (Fig. 1).

The first report demonstrating functional coupling of a heterologous GPCR to the yeast mating pathway, i.e. human β2-adrenergic receptor, suggested a significant conservation of the GPCR-machineries of mammalian and yeast cells [12].

In the present work we aimed to use the particular advantages of the yeast mating pathway system to study human Frizzled receptors. Support for this approach recently came from the whole genome sequence analysis of the social amoeba *Dictyostelium discoideum*, which identified predicted receptors of the frizzled/smoothened class (family 5) in this organism. Previously, such receptors were thought to be a domain of higher animals [13]. As *Dictyostelium* represents an evolutionary older organism than yeast, Frizzleds may be considered as archetypic receptors, suggesting that they may be compatible with the yeast GPCR- machinery.

We show that both human Frizzled1 and Frizzled2, considered as canonical and noncanonical receptors, respectively, can signal to the yeast mating pathway in the absence of added ligand. Overexpression of Frizzled receptors leads to increased accumulation of cells in the G1 phase of the cell cycle, a feature of the activated mating pathway, and activation of a specific mating pathway reporter. We devised specific receptor chimeras between Frizzleds and the yeast α-factor receptor Ste2p for pharmacological studies with Wnt-ligands and α-factor pheromone, and identify chimeras mediating highly penetrant G1-phase arrests. These effects are partially reversible by deletion of the mating pathway effector gene *FAR1* [14].

## Results

### Expression of human Frizzled receptors in yeast

**Figure 2:**
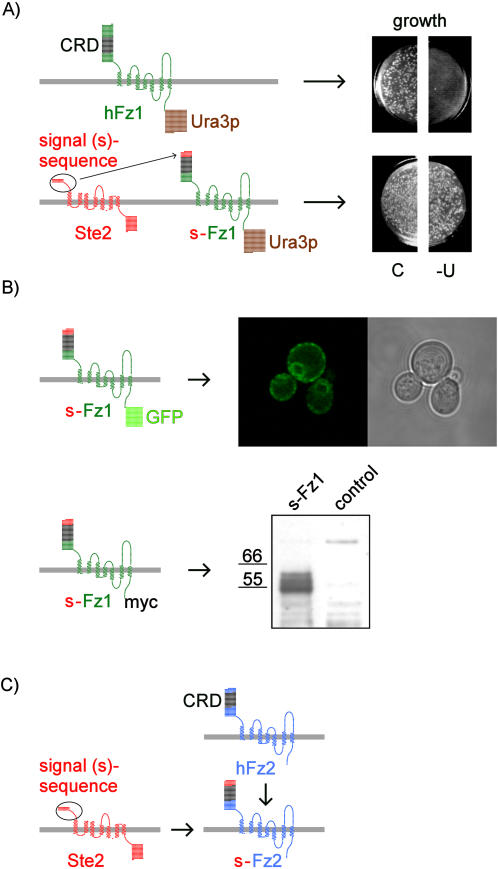
Expression strategy and expression tests for human Frizzled receptors in yeast. A. Expression growth test for Fz1 using the nutritional reporter Ura3p. Schematically shown as two-dimensional snake-plots are Fz1, or Fz1 with the N-terminal signal sequence (s) of the α-factor receptor Ste2p fused upstream of the cysteine-rich-domain (CRD), and carrying a C-terminal Ura3p reporter. The constructs were used to transform *URA3*-deficient yeast cells, and plated equally onto control plates (C) and minimal medium plates lacking uracil (-U). B. Subcellular localization and Western blot analysis. The upper panel shows a confocal micrograph and the corresponsing bright field view of s-Fz1-GFP expressing yeast cells immobilized by agarose-embedding. The lower panel shows the Western blot analysis using s-Fz1-myc expressing yeast cells. Following galactose-induction, crude protein extracts were prepared and resolved on a 4–12% gradient SDS-PAGE system. Detection was performed using an anti-c-myc monoclonal antibody-peroxidase conjugate. Molecular weight standards in kDa are indicated. The control extract was derived from a yeast strain transformed with the empty expression vector. C. The s-Fz2 construct for the expression of human Frizzled 2 was analogous to s-Fz1.

A previous study, using a topology-sensitive reporter, suggested that the *Drosophila* Frizzled receptor expressed in yeast has the structure of a seven-pass transmembrane protein [15]. We first explored the feasibility to express human Fz1 in a yeast wildtype strain by a related approach: We took advantage of the nutritional reporter Ura3p fused to the C-terminus of Fz1 as shown in Fig. 2A. The *URA3* gene encodes orotidine-5′-phosphate decarboxylase which functions in the biosynthesis of pyrimidines in the cytosol. Used as a fusion tag it provides information on the fate of the tagged protein. Absence of growth on minimal media lacking uracil indicates insufficient cytosolic Ura3p levels, which may result from improper protein synthesis or transport. Yeast expressing our initial Fz1-Ura3p construct failed to grow in the absence of uracil (Fig. 2A, top), and we reasoned that this might be the result of improper targeting of the protein to the yeast secretory pathway. To circumvent potential problems with the N-terminal sorting information of the mammalian Fz1, we devised a chimeric receptor consisting of human Fz1 with the amino acids N-terminal to the cysteine-rich domain (CRD) replaced with the extracellular N-terminal tail from the yeast α-factor receptor Ste2p (termed signal sequence “s” in the following). This design was chosen to preserve the Wnt-ligand binding region that is attributed to the CRD, a characteristic feature of Frizzled receptors [16].

As shown in Fig. 2A (bottom), the growth assay using Ura3p fused to the C-terminus of the s-Fz1 chimeric receptor indicates good expression - colony numbers and growth rates are comparable to the control plate. This is consistent with proper membrane insertion of the receptor with the Ura3p moiety facing the cytosolic space.

In order to further clarify the subcellular localization of the s-Fz1 receptor, we fused enhanced green fluorescent protein (eGFP) to the receptor's C-terminus to be able to monitor expression using confocal microscopy. As shown in Fig. 2B, we found a pronounced plasma membrane rim-like GFP signal, as well as apparently perinuclear staining which is similar to the localization described previously for GFP-tagged Ste2p [17].

In Western blot analysis using a myc-tagged version of the s-Fz1 receptor we obtained a signal of expected size (60 kDa). The broad appearance of the band points to the glycosylated status of the protein, which is a frequent modification of serpentine receptors (Fig. 2B).

In addition to the ‘canonical’ Frizzled 1 receptor, we were interested in studying the ‘noncanonical’ Frizzled 2 receptor in the yeast system. We constructed the s-Fz2 receptor construct in analogy to s-Fz1 as depicted in Fig. 2C. Both receptors showed similar functionality in yeast (see below).

### Growth modulatory effects and activation of a mating pathway reporter system

**Figure 3:**
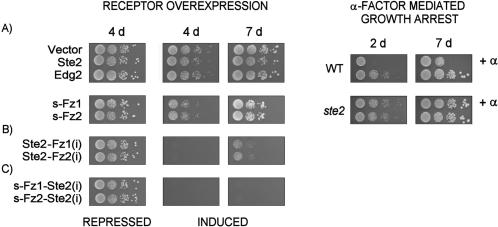
Growth modulation assay of wildtype yeast cells (MH272-1a) overexpressing human Frizzled receptors and receptor chimeras. s-Fz1 and s-Fz2 (panel A) as well as receptor chimeras with the Ste2 receptor (B and C) were expressed in a galactose-inducible system, and effects were compared to α-factor mediated growth arrest. Corresponding receptor schemes are shown in Fig. 2 and Fig. 5. Yeast strains transformed with plasmid constructs as indicated were pre-cultured in glucose-containing minimal medium (repressed), and spotted in serial dilutions (∼1E4, 1E3, 1E2, 1E1 cells) onto glucose- or galactose- (induced) containing minimal medium agar plates selective for the presence of the plasmid. Growth effects were recorded after 4 and 7 days. Controls were the empty expression vector and Ste2 and Edg2 receptors. The α-factor - mediated growth arrest assay (right panel) was done using wildtype yeast cells (MH272-1da), as well as isogenic *ste2* cells, treated with 1 µg α-factor spotted directly onto the yeast patches as indicated.

In growth experiments with wildtype yeast, we consistently found that the overexpression of both s-Fz1 and s-Fz2 receptors in a galactose-inducible system (*GAL1* promoter) leads to a weak but significant growth inhibition (Fig. 3A). Absence of such effects using overexpressed Ste2p, or the human Edg2 receptor, that is known to couple to Gpa1p in yeast [18], indicates the specificity of the Frizzled-mediated growth inhibition (Fig. 3A). Mating pathway activation by α-factor induced growth arrest occured as expected (Fig. 3A, right panel).

We next set out to confirm the basal activities of s-Fz1/2 observed in the growth modulation assays using a specific mating pathway reporter system. This system employs a firefly luciferase reporter gene under the control of the pheromone-responsive *FUS1* promoter (P*_FUS1_*-luciferase; Fig. 1). We used this reporter construct in a specifically engineered yeast strain background that carries a *gpa1* allele (termed MC18; [19]). The system allows the use of plasmid - encoded heterologous Gα proteins to replace the yeast Gα Gpa1p. In the absence of exogenous Gpa1p this strain shows constitutive mating pathway activation, and we call it “ON” strain in the following. Conversely, expression of Gpa1p in the absence of any regulating GPCR fully suppresses the mating pathway (“OFF” strain). Previously, the MC18 system was used successfully to study the coupling of olfactory GPCRs to the mating pathway [20], [21]. For the present study we introduced two additional deletions into the MC18 strain background: We first carried out a *STE2* deletion to be able to test pathway activation independently of the endogenous Ste2 receptor (termed MC18 *ste2*), and secondly we introduced a deletion of the negative pathway regulator *SST2* (MC18 *ste2 sst2*) in order to obtain a potentially more sensitive strain ([10]; shown schematically in Fig. 1).

**Figure 4:**
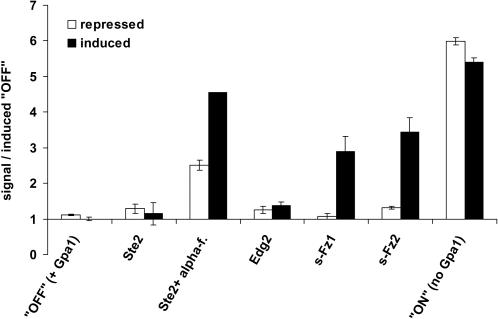
Effect of galactose-inducible overexpression of human Frizzled receptors (s-Fz1 and s-Fz2) in the P*_FUS1_*-luciferase- based reporter system. Basal activities of s-Fz1 and s-Fz2 in absence of added ligand were determined. Schemes for receptor constructs used are shown in Fig. 2. Yeast strain MC18 *ste2 sst2* expressing plasmid-encoded Gpa1p was employed. Crude extracts from glucose-repressed or galactose-induced yeast strains were assayed in duplicates for luciferase activity. Normalization was done based on total protein concentration. Signals are expressed relative to the receptor-deficient “OFF”-strain grown under induced conditions. Ste2p- (treated and untreated with 1 µM α-factor), Edg2-overexpressing strains, as well as the *GPA1*-deficient “ON”-strain were included as further controls. Bars represent average+/−range.

We used the P*_FUS1_*-luciferase reporter system to determine the mating pathway activation status following overexpression of the Frizzled proteins in absence of added ligand. To this end, reporter gene activity was determined under conditions of glucose-repression and galactose-induction. As shown in Fig. 4, both s-Fz1 and s-Fz2 display significant inducible activity over the OFF-strain. The three to four - fold induction levels observed are comparable to peak-signaling levels for the α-factor - activated Ste2-receptor (Fig. 4). Overexpression of the Ste2 or Edg2 receptors alone does not raise the signal significantly, again demonstrating specificity of action of the Frizzled receptors. For the *GPA1*-deficient ON-strain an approximately 5-fold induction over the OFF-strain is observed, as expected. This is consistent with peak βγ signaling in absence of any Gpa1p. In conclusion, these data are in line with the observations in the growth modulation assays and demonstrate signaling of the s-Fz1 and s-Fz2 receptors to the yeast mating pathway.

We were unable to demonstrate further activation of the mating pathway with commercially available bioactive recombinant Wnt3a and Wnt5a (data not shown). This may be due to the lack of appropriate co-receptors in yeast, or the inability of these ligands to pass through the cell wall (see discussion).

### Receptor chimeras between Ste2p and Frizzled 1 or Frizzled 2

**Figure 5:**
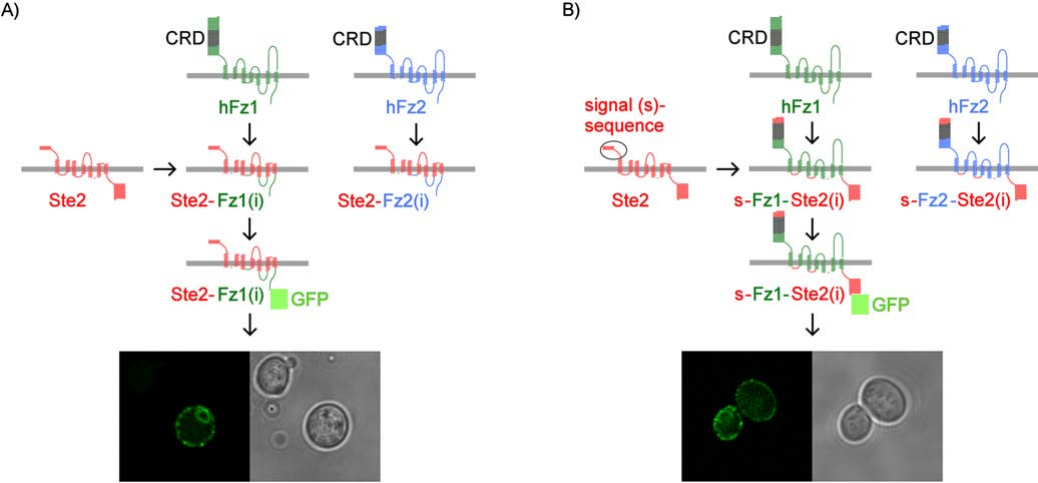
Construction schemes and subcellular localization of chimeras between the human Frizzled and *S. cerevisiae* Ste2 receptors. Receptor homology modeling was used to predict the transmembrane segments (see Fig. 6). A. This chimera type replaces the predicted intracellular portions of Ste2p with the corresponding ones of either Fz1 or Fz2, resulting in chimeras termed Ste2-Fz1(i) and Ste2-Fz2(i), respectively, with “(i)” designating intracellular portions. B. The second chimera type replaces the predicted intracellular portions of the human Frizzled receptors with respective ones of Ste2p, and uses the signal sequence of Ste2p (“s”) upstream of the cysteine-rich-domain (CRD) to give rise to s-Fz1-Ste2(i) and s-Fz2-Ste2(i). The fully synthetic gene sequences and respective amino acid sequences are given in the *Supporting Information* section of this article (Text S1). The subcellular localization of constructs tagged C-terminally with GFP is shown in the confocal micrographs.

**Figure 6:**
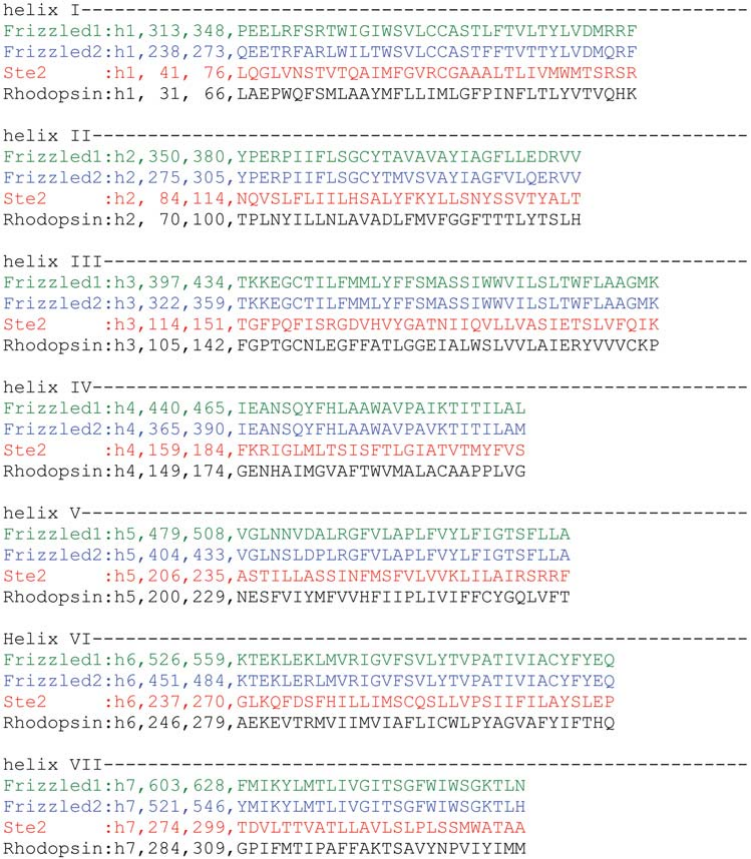
Transmembrane segment predictions for human Fz1, Fz2, and *S. cerevisiae* Ste2 receptors. The helices one to seven are shown as amino-acid sequences with indicated position numbers of the full sequence. The predictions were derived by receptor modeling. The transmembrane region assignment was based on a hydrophobicity analysis followed by manual adjustment of the overlap of some key amino acids (e.g., W and P in TM4, TM5, and TM6) with those found in rhodopsin and other reference receptors.

In analogy to work performed previously [7], [22] we set out to engineer two types of chimeras between the yeast Ste2 receptor and the human Frizzled proteins. First we replaced predicted intracellular amino acids of Ste2 with corresponding fragments of Frizzled receptors, with the aim to demonstrate that the Frizzled sequences would be compatible with activation of Gpa1p. We also hoped to retain responsiveness of these constructs to α-factor. Conversely, by introducing intracellular Ste2 loops into the Frizzled backbone, we aimed to increase the functional coupling of the Frizzled receptors to Gpa1p. The receptor chimeras are shown in Fig. 5, and the nucleotide and amino acid sequences of the fully synthetic genes are shown in the *Supporting Information* section of this article (Text S1). The underlying transmembrane region assignments for the Fz1, Fz2 and Ste2 receptors (Fig. 6) were derived by receptor homology-modeling.

The first constructs were named Ste2-Fz1(i) and Ste2-Fz2(i), respectively, with “(i)” designating the intracellular portions of the Frizzled receptors. The inverse constructs are named s-Fz1-Ste2(i) and s-Fz2-Ste2(i), respectively.

**Figure 7:**
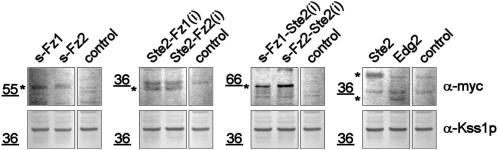
Quantification of receptor expression by Western blot. Following galactose-induction, crude protein extracts were prepared from the MH272-1da wildtype yeast strain transformed with the indicated plasmids, and resolved on a 4–12% gradient SDS-PAGE system. Detection of the myc-tagged receptors was performed using chromogenic indirect anti-c-myc monoclonal detection. Equal loading was monitored using a polyclonal anti-Kss1p antibody. The control extract was derived from a yeast strain transformed with the empty expression vector. The asterisks indicate the specific signals. Molecular weight standards in kDa are indicated.

First we tested the expression and subcellular localization of chimeras by fusing GFP to the C-termini. Confocal microscopy demonstrated the expected plasma membrane localization similar to s-Fz1-GFP (Fig. 5A), supporting the validity of the homology models employed to design the constructs. In Western blot analysis using myc-tagged versions of the chimeras we observed signals of expected size (both ∼37 kDa; Fig. 7). The expression level of the Ste2p receptor, and the Ste2 - Frizzled 1/2 receptor chimeras expressed from the codon-optimized synthetic genes were slightly higher than for the natural human genes s-Fz1/2 and Edg2. However, the Ste2p overexpression demonstrates that high protein levels alone do not translate into a growth phenotype.

**Figure 8:**
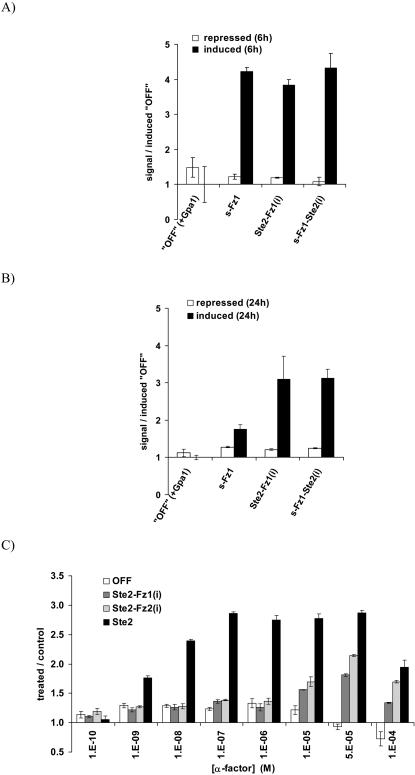
Effect of galactose-inducible overexpression of the receptor chimeras between human Frizzled receptors (Fz1 and Fz2) and the *S. cerevisiae* Ste2 receptor in the P*_FUS1_*-luciferase- based reporter system. Yeast strain MC18 *ste2 sst2* expressing plasmid-encoded Gpa1p was employed. Schemes for receptor constructs used are shown in Fig. 5. A. and B. Basal activities of Ste2-Fz1(i) and s-Fz1-Ste2(i) receptors in absence of added ligand, and comparison to the s-Fz1 overexpressing strain. Crude extracts from glucose-repressed or galactose-induced yeast strains were assayed in duplicates for luciferase activity after 6 h (A.) or 24 h (B). Normalization was done based on total protein concentration. Signals are expressed relative to the receptor-deficient “OFF”-strain grown under induced conditions. Bars represent means+/−range. C. α-factor concentration - response in the MC18 *ste2 sst2* strain expressing plasmid-encoded Gpa1p and Ste2-Fz1/2(i) receptor chimeras or Ste2p. The galactose-induced yeast strains were incubated for 4 hours with indicated concentrations of α-factor, and crude extracts were subsequently assayed in quadruplets for luciferase activity. As negative control served a strain transformed with empty vector (“OFF”-strain). Responses are expressed as treated/control values; error bars indicate S.E.M. (N = 4).

Interestingly, in growth experiments (Fig. 3B), the Ste2-Fz1/2(i) receptor chimeras exerted strong growth arrest in the absence of added α-factor, and the effects were stronger than observed for the Frizzled constructs described before (s-Fz1/2; Fig. 3A). This suggests that the intracellular portions of both Fz1 and Fz2 are able to contact the yeast Gα protein Gpa1p. Indeed, using the P*_FUS1_*-luciferase reporter system we observed significant inducible activity over the OFF-strain for the overexpressed Ste2-Fz1(i) receptor, similar to both native s-Fz1 (Fig. 8A), and to α-factor - activated Ste2-receptor (Fig. 4).

We next tested whether the Ste2-Fz1/2(i) type chimeras were still able to respond to α-factor. As is shown in Fig. 8C this is indeed the case, however, the potency of the response is significantly lower for these chimeras than for the native Ste2 receptor. In concentration - response experiments we estimated EC50 values for both Ste2-Fz1/2(i) in the low micromolar range versus a low nanomolar range for Ste2p, which is in agreement with literature data [23]. The signals were consistently above those obtained for the receptor-deficient OFF-strain.

When we tested the s-Fz1/2-Ste2(i) chimeras in the growth modulation assay, we observed that both receptors of this type exert a complete growth arrest (Fig. 3C), even more pronounced than the α-factor response in wild type (Fig. 3A, right panel). This effect is consistent with the Ste2p-portions mediating contact to the yeast Gα protein Gpa1p, and acting cooperatively with the Frizzled receptor backbone to adopt an active conformational state. Expectedly, upon overexpression of the s-Fz1/2-Ste2(i) chimeras in a yeast wildtype strain this growth behaviour was paralleled by significant inducible activity over the OFF-strain in the P*_FUS1_*-luciferase reporter system, similar to what was observed for native s-Fz1 and the Ste2-Fz1(i) chimera (Fig. 8A). Peak signaling levels were as observed for the α-factor - activated Ste2 receptor (Fig. 4).

The P*_FUS1_*-luciferase reporter system did not differentiate quantitatively between the native s-Fz1/2 and chimeric receptor constructs as observed in the growth assay (compare Fig. 8A and Fig. 3). Since a major difference between these two experimental readouts is the time following induction of receptor expression (6 hours for the P*_FUS1_*-luciferase reporter system vs. up to 7 days for the plate growth assay), we decided to measure P*_FUS1_*-luciferase activity after prolonged incubation (24 hours). In better agreement with the long-term growth assays, we observed strong persistent signaling for the chimeric receptors Ste2-Fz1(i) and s-Fz1-Ste2(i) after 24 hours, whereas the signal for the s-Fz1 construct was reduced. (Fig. 8B). The reason for this apparently differential desensitization is not known.

### Growth in liquid culture and budding index

**Figure 9:**
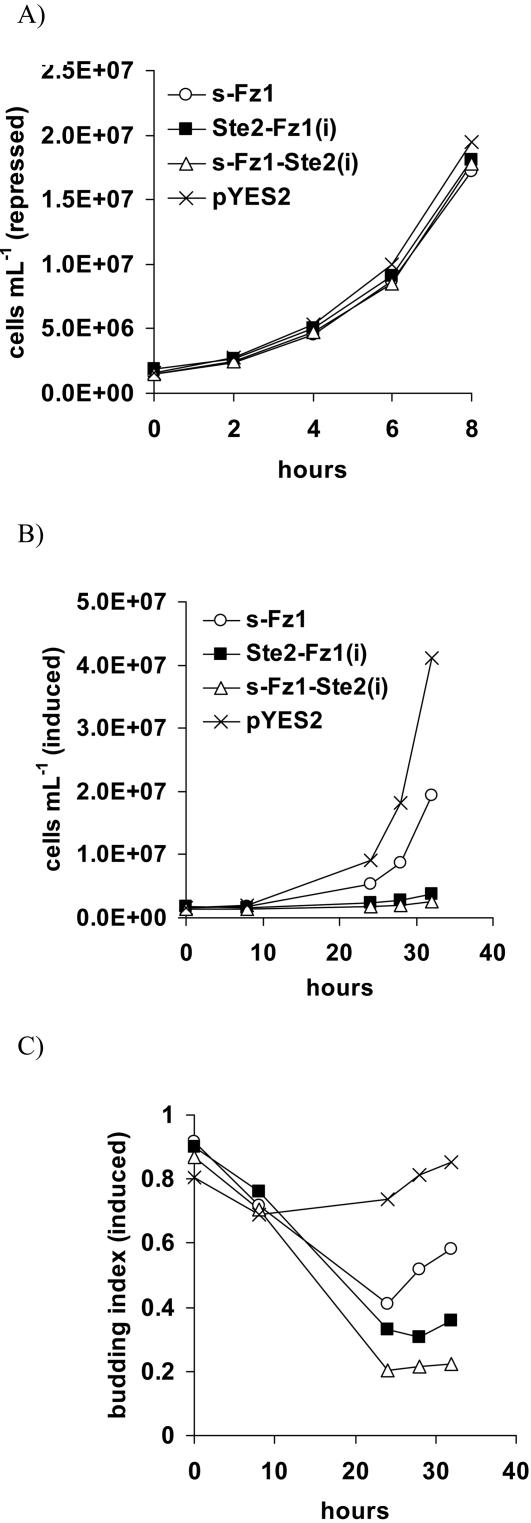
Growth behaviour and morphologies of wildtype yeast cells (MH272-1da) overexpressing galactose-inducible s-Fz1 or receptor chimeras between Fz1 and Ste2p in liquid culture. Schemes for receptor constructs used are shown in Fig. 2 and Fig. 5. As control served a strain transformed with empty vector. Growth curves in glucose- (repressed; (A)), or galactose-containing (induced; (B.)) minimal medium, following washout of glucose, as determined using optical density measurement, or microscopic counting in a hemocytometer slide at the indicated timepoints, respectively. C. Budding indices (ratio of budded to total yeast cells) of the induced cultures were determined using microscopic counting in a hemocytometer slide.

Cell cycle arrest following mating pathway activation results in an “unbudded” cellular morphology, and eventually induction of a “shmoo”- morphology prior to diploid formation. Therefore, we were interested to investigate the morphological changes of a yeast wildtype strain following galactose-induction of s-Fz1, Ste2-Fz1(i) or s-Fz1-Ste2(i) receptor expression in liquid culture. For this purpose, we first monitored the relative growth rates of the strains grown in repressing or inducing medium. The result was consistent with the plate growth assay (Fig. 3): In the absence of receptor expression the three strains grew comparably to the empty-vector control strain (Fig. 9A). When switched to inducing medium, following a lag-phase due to adaptation, the strains grew differentially with the s-Fz1 strain showing significant growth retardation, and the strains carrying the chimeric receptors showing almost complete growth arrest. (Fig. 9B). Consistently with a differential ability of the three receptor constructs to induce G1 arrest, we determined significantly lowered budding indices down to ∼0.4 for the s-Fz1 strain, ∼0.3 for Ste2-Fz1(i) and ∼0.2 for s-Fz1-Ste2(i) after 24 hours grown in the inducing medium (Fig. 9C). Moreover, while the s-Fz1 expressing strain was able to recover from the G1 arrest after 24 hours, as indicated by an increased budding index up to ∼0.6, the receptor chimeras appeared to be permanently arrested. This is in agreement with the reduced P*_FUS1_*-luciferase activity measured 24h after receptor induction (Fig. 8B). Specific G1 arrests we further confirmed by investigating the nuclear morphologies using staining by 4′,6-diamidino-2-phenylindole (data not shown). However, we did not observe “shmoo”-like shaped cells at any time point upon overexpression of any of the three receptor classes. This is similar to previous observations made for constitutively active mutants of Ste2p that showed only weak induction of projections despite high P*_FUS1_*-reporter levels [24].

### Suppression of Frizzled-mediated growth arrest by deletion of the mating pathway effector *FAR1*

**Figure 10:**
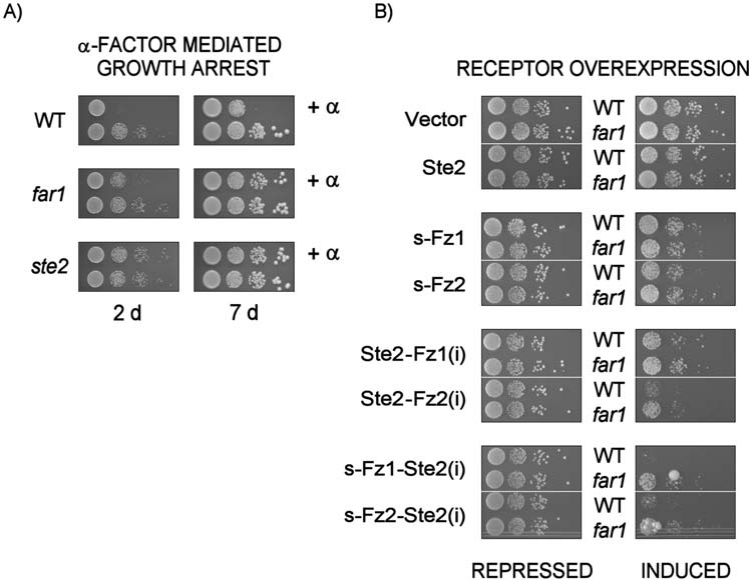
Mating pathway activation growth assay of wildtype yeast cells (MH272-1da), as well as of the isogenic strains carrying *far1* or *ste2* - alleles. (A) Yeast strains were spotted in serial dilutions (∼1E4, 1E3, 1E2, 1E1 cells in duplicate) onto complete minimal medium, and treated with 1 µg α-factor spotted directly onto the yeast patches. Growth effects were recorded after 2 and 7 days. (B) Comparative growth modulation assay of wildtype yeast cells (MH272-1a) and respective *far1* isogenic strain overexpressing human Frizzled receptors (s-Fz1 and s-Fz2), as well as chimeras with the *S. cerevisiae* Ste2 receptor in a galactose-inducible expression system. The corresponding receptor schemes are shown in Fig. 2 and Fig. 5, respectively. Yeast strains transformed with plasmid constructs as indicated were pre-cultured in glucose-containing minimal medium (repressed), and spotted in serial dilutions (∼1E4, 1E3, 1E2, 1E1 cells) onto glucose- or galactose- (induced) containing minimal medium agar plates selective for the presence of the plasmid. Growth effects were recorded after 4 to 7 days. Controls were the empty expression vector, as well as overexpressed Ste2 and Edg2 receptors.

In order to further exclude growth inhibition by unspecific effects, we aimed to demonstrate genetically that the growth phenotypes conveyed by Frizzled expression are dependent on a functional mating pathway. The *FAR1* gene product is required for growth arrest in the G1 phase of the cell cycle following mating pathway activation. Inactivation of the *FAR1* gene leads to cell cycle progression despite activated mating pathway through antagonizing the cyclin Cln2p (Fig. 1) [14]. Therefore, we reasoned that the deletion of the *FAR1* gene would result in suppression of the growth effects seen for the receptor chimeras between Ste2p and Fz1/2. We created a *far1* yeast strain and tested first its responsiveness to α-factor in a growth assay compared to its parent wildtype strain, as well as an isogenic *ste2* strain (Fig. 10A). The expected growth arrest is observed for the wildtype strain in the presence of α-factor, whereas the *far1* strain shows the anticipated progression to the cell cycle, and unchanged survival upon prolonged incubation (7 days). The partial suppression of the mating pathway-induced growth arrest seen in the *far1* strain after short incubation (2 days) is consistent with the functional redundancy of Cln2p with the two other cyclins functional in yeast [14]. Expectedly, the *ste2* strain shows no response to α-factor (Fig. 10A).

The differential growth behavior test of wildtype and *far1* strains overexpressing the Frizzled receptors and respective chimeras with Ste2p under galactose-induction is shown in Fig. 10B. The empty-vector control demonstrates that both strains have the same growth behavior both on glucose- and galactose containing medium. As anticipated, all three chimeric receptor classes show a clear gain-of-growth phenotype in the presence of the *far1* strain as compared to the wildtype strain. The only exception is the s-Fz1 receptor, which exerted only very weak growth modulatory effects in this experiment. The *far1* effect is particularly obvious in the case of the s-Fz1/2-Ste2(i) chimeras, where complete growth arrest is observed under induced conditions for the wildtype strain. As expected, no effect of the *far1* allele was observed in strains overexpressing Ste2p. These results confirm that the growth arrest effects seen for the overexpressed receptor chimeras depend on the activated mating pathway.

## Discussion

Signaling of human Frizzled receptors to the yeast mating pathway is particularly interesting from the evolutionary perspective: Our results suggest that the archetypic GPCR signal transduction machinery of the yeast *S. cerevisiae* may be related to Frizzled signaling in animals. It is therefore also conceivable that the recently identified Frizzled-like receptors in the genome of the evolutionary older organism *Dictyostelium* [13] function through a similar ancestral mechanism.

Previous work has provided some evidence that Frizzled receptors could act as G protein-coupled receptors [4], [5]. However, commonly used functional tests for GPCRs in mammalian cells (e.g. modulation of adenylyl cyclase and phospholipase C activities) could not be applied successfully to Frizzled receptors. In this work, we have attempted to use yeast as a model organism to study Frizzled function. We show that overexpressed human Frizzled 1 and Frizzled 2 receptors partially induce G1 cell cycle arrest in wildtype yeast cells, a feature of the activated mating pathway, and significant P*_FUS1_*-dependent reporter gene induction.

Specific receptor chimeras between Fz1/2 and the yeast α-factor receptor Ste2p showed that the predicted intracellular portions of Fz1/2 are sufficient to signal to the mating pathway if incorporated into Ste2p, as indicated by a highly penetrant G1 cell cycle arrest, and P*_FUS1_*-reporter activity. Moreover, the effect on growth arrest and ability to induce P*_FUS1_*-reporter activity seen for Ste2-Fz1(i) and Ste2-Fz2(i) were almost indistinguishable, which is in line with the proposed receptor model (Fig. 6): Despite their different modes of signaling reported in higher eukaryotes (canonical vs. non-canonical; [25], [26], and [22], [27], [28], respectively), the predicted intracellular portions of Fz1 and Fz2 differ by only three amino acids. The responsiveness of these chimeras to α-factor further supports their functionality as GPCRs signaling to yeast Gpa1p.

Strong growth arrest paralleled with P*_FUS1_*-reporter activity was also observed for the converse receptor chimeras, s-Fz1/2-Ste2(i), which employ the Ste2-intracellular portions, expected to afford good coupling to yeast Gpa1p. Overexpressed native Ste2-receptor does not show any growth effect in the absence of added α-factor, demonstrating that the Fz1 and Fz2 transmembrane scaffolds allow the chimeras to adopt a conformationally active state.

Indeed, cooperative action between the transmembrane segments and connecting loops for establishing the three-dimensional structure has been described for several GPCRs including Ste2p [29]: Specific receptor fragments were capable of intragenic allelic complementation in the absence of a covalent connection of the transmembrane segments. These data support a two-stage model of GPCR folding, in which the transmembrane α-helices are first formed independently, and then are assembled into tertiary structures.

We were unable to demonstrate Wnt-dependent activation of s-Fz1/2 receptors, or the s-Fz1/2-Ste2(i) chimeras, in yeast using the P*_FUS1_*-luciferase system. Different reasons may account for this result. First, the basal activities observed for both receptor classes were close to the maximal response observed for the α-factor-activated native Ste2 receptor, hence the response may be already saturated. On the other hand we observed α-factor responsiveness of the Ste2-Fz1/2(i) receptor constructs despite similar basal activity, albeit at significantly lower potency as compared to native Ste2p. More importantly, at least for the canonical Frizzled 1, the LRP5/6 co-receptors appear crucial for Wnt ligand binding and signaling in mammalian cells (reviewed in [3]). Yeast does not express known co-receptors of this type, such molecules may have to be supplemented in the heterologous yeast system as well. Additionally, despite the fact that yeast cells can secrete proteins of >200 kDa [30], it is generally believed that proteins up to 5–10 kDa can pass through the cell wall. Consequently, the lipid-modified Wnt-ligands (approximately 40 kDa) may not pass through the yeast cell wall to reach the receptors. A possible solution to this problem may be the use of an autocrine Wnt-Fz activation system, similarly as described for the autocrine activation of the C5a receptor through an α-factor prepro/C5a ligand [31]. Another possible solution would be to examine spheroplasts or mutants with increased cell wall permeability.

Constitutive activity has been described for many wildtype GPCRs coupled to G_s_-, G_i_ and G_q_-proteins (reviewed in [32]). It is frequently unmasked by recombinant systems overexpressing receptors, as is the case in the present study using yeast. Constitutive activities of GPCRs are also known to depend on cellular background [32] as cell types may express a different G protein repertoire and different regulatory proteins. To our knowledge, constitutive activity of native Frizzled receptors has not been addressed in the published literature, which is in part due to a lack of convenient pharmacological tools.

Ste2p receptor oligomerization is thought to promote receptor biogenesis, G protein signaling and endocytic trafficking [33], and functionally relevant hetero-oligomerization of GPCRs has been demonstrated in higher organisms, notably for GABA(B)R1 and GABA(B)R2 receptor subunits [34]. While it is formally possible that Ste2 - Fz hetero-oligomerization contributes to the growth effects seen in wildtype yeast cells, we observed autonomous constitutive activity of the Frizzled receptor constructs in our *STE2*-deficient P*_FUS1_*-dependent reporter gene system.

In yeast, genetic screens were used to isolate mutations that constitutively activate the Ste2p receptor [24], [35]. Interestingly, similar features to the present constitutively active receptors have been described: This includes the observation that the overexpressed Ste2-P258L, S259L -mutant receptor shows P*_FUS1_*-reporter levels comparable to the maximal response for α-factor-activated native Ste2p [24], despite only weak induction of the expected “shmoo”-morphology. This has been attributed to the potential inability of these constitutively active receptors to stimulate receptor functions in morphogenesis that are not mediated by G protein activation [36], [37].

To demonstrate that the growth arrest effects seen for the constitutively active Ste2 - Fz 1/2 receptor chimeras are a consequence of mating pathway activation we have used a genetic approach: Deletion of the cyclin-dependent kinase inhibitor *FAR1* partially reversed the growth arrest phenotypes. Partial reversal was expected, as there are *FAR1 - CLN2* independent parallel pathways to mediate growth arrest in wild type yeast cells [14].

Coupling of Frizzled receptors to the yeast mating pathway parallels earlier observations with several mammalian GPCRs expressed in this organism [38], [39]. Receptors coupling to various mammalian G proteins (G_s_-, G_i_ and G_q_) could be functionally expressed, suggesting a considerable degree of evolutionary conservation at the GPCR - Gα-protein interface. However, attempts to clearly define GPCR - elements of the primary or secondary structure responsible for Gα-coupling specificity have not been successful. This phenomenon is particularly well illustrated by the yeast Ste2 and Ste3 receptors: Despite low sequence identity (11%), both couple to the same Gα protein, Gpa1p. Among the four mammalian Gα - subfamilies the G_i_-subfamily shares the closest sequence similarity with yeast Gpa1p. This subfamily encompasses G_o_ and G_t_, which have been proposed previously to be involved in Frizzled 1 and Frizzled 2 signaling, respectively ([25], [26], and [22], [27], [28]).

In summary, our report demonstrates that human Frizzled receptors can signal to the mating pathway in yeast, and this coupling can be made particulary efficient by introducing intracellular sequence elements of the Ste2 mating factor receptor into the Frizzled backbone. This experimental system may prove useful for the identification and characterization of modulators of individual Frizzled receptors.

## Material and Methods

### *E. coli* and *S. cerevisiae* strains

All plasmid constructions and amplifications were performed using the *E. coli* strains XL1-Blue (Stratagene) or TOP10 (Invitrogen). For the expression growth test of the Ura3p fusion proteins (Fz1-Ura3p and s-Fz1-Ura3p, see below) yeast strain JD53 *MATα his3-Δ200 leu2-3,112 lys2-801 trp1-Δ63 ura3-52* was used [40]. For confocal microscopy and Western blot analysis the yeast strain MH272-1da *MAT* ***a*** *his3 leu2-3,112 ura3-52 trp1 rme1 HMLa;* [41]) was used. For the growth modulation assays MH272-1da and respective isogenic strains MH272-1da *ste2::hphMX4 and MH272-1da far1::hphMX4* were used (this study, see below). The mating pathway activation P*_FUS1_*-luciferase assays were carried out using the MC18 *ste2::hphMX4 sst2::kanMX4* strain (this study, see below), a descendant of the previously described strain MC18 *MAT* ***a*** *gpa1::lacZ[LEU2]ade2-1 his3-11, 15 leu2-3112 trp1-1 ura3-1 can1–100*; [19].

MH272-1da *ste2::hphMX4*: The hygromycin phopsphotransferase disruption cassette was amplified by PCR using the *STE2*-targeting primers: DD_46 (5′-GCTCTGGCTATAATTATAATTGGTTACTTAAAAATGCACCGTTAAGAACCATATCCAAGAATCCAGCTGAAGCTTCGTACGCTGCAGG-3′) and DD_47 (5′-CTAGTAGTAACCTTATACCGAAGGTCACGAAATTACTTTTTCAAAGCCGTAAATTTTGGCCGCATAGGCCACTAGTGGATCTG-3′). These primers were derived from the plasmid construct pAG32 [42]. The resulting PCR-product was used to transform the MH272-1da strain that was plated onto YPD plates containing 300 µg mL^−1^ hygromycin B (Sigma). Correct integration of the cassette into the *STE2* locus was verified by colony-PCR using primers DD_42 (5′-CTGCTTAGGACCTGTGCCTGGA-3′) and DD_43 (5′-GCTTGAACTCGTAAAAGCAAAG-3′) flanking the Ste2p coding sequence. Following cloning of the PCR product into pCR4-TOPO (Invitrogen), sequencing confirmed the correct integration.

MH272-1da *far1::hphMX4*: The hygromycin phopsphotransferase disruption cassette was amplified by PCR using the *FAR1*-targeting primers: DD_84 (5′-CCTTTACACAAAGTCTATAGATCCACTGGAAAGCTTCGTGGGCGTAAGAAGGCAATCTATTACAGCTGAAGCTTCGTACGCTGCAGG-3′) and DD_87 (5′-CACCCGCAGCCATATCCCCCAAATATACTTGCTCGAATGTAGCTTGTGGTAGACGAAGAGGCCGCATAGGCCACTAGTGGATCTG-3′). These primers were derived from the plasmid construct pAG32 [42]. The resulting PCR-product was used to transform the MH272-1da strain that was plated onto YPD plates containing 300 µg mL^−1^ hygromycin B (Sigma). Correct integration of the cassette into the *FAR1* locus was verified by colony-PCR using primers DD_86 (5′-gttcactctgtcttgagagtg-3′), priming in the 5′ UTR of the *FAR1* locus, and DD_72 (5′-ccatggcctccgcgaccgg-3′) priming in the disruption cassette. Following cloning of the PCR product into pCR4-TOPO (Invitrogen), sequencing confirmed the correct integration.

MC18 *ste2::hphMX4 sst2::kanMX4*: The targeted disruptions of *STE2* and *SST2* genes were performed sequentially as follows. The disruption of *STE2* was performed as described for MH272-1da *ste2::hphMX4* except that MC18 was employed as parent strain. The resulting strain was termed MC18 *ste2::hphMX4*. For subsequent disruption of *SST2*, the kanamycin resistance disruption cassette was amplified by PCR using primers DD_68 (5′-GTTTTGCACGCACTATCTGAGGCGTTATAGGTTCAATTTGGTAATTAAAGATAGAGTTGTAAGCAGCTGAAGCTTCGTACGCTGCAGG-3′) and DD_69 (5′-CTAAAGAAAAAAAAAAGGACTGTTTGTGCAATTGTACCTGAAGATGAGTAAGACTCTCAATGGCCGCATAGGCCACTAGTGGATCTG-3′) derived from pFA6a-kanMX4 [43]. The resulting PCR-product was used to transform the MC18 *ste2::hphMX4* strain that was plated onto YPD plates containing 300 µg mL^−1^ hygromycin B (Sigma) and 200 µg mL^−1 ^geneticin (Invitrogen).

Correct integration of the cassette into the *SST2* locus was verified by colony-PCR using primers DD_70 (5′-GTCTTTACTTCTCATCGTCC-3′), priming in the 5′ UTR of the *SST2* locus, and DD_71 (5′-GCGCATCGGGCTTCCCATAC-3′) priming in the disruption cassette. Following cloning of the PCR product into pCR4-TOPO (Invitrogen), sequencing confirmed the correct integration. The resulting strain was termed MC18 s*te2::hphMX4 sst2::kanMX4*.

### Plasmid constructs

Correctness of all constructs was determined using sequencing. Below described constructs pP*_CUP1_*-Fz1-CRU and pP*_CUP1_*-s-Fz1-CRU are derived from a parallel study to search for protein-protein interactions of the Frizzled 1 receptor using the Ura3p-based yeast split-ubiquitin interaction system [44] that will be published separately (Dirnberger D. and Baumeister R., University of Freiburg, Germany; manuscript in preparation).

pP*_CUP1_*-Fz1-CRU: This construct is based on the previously described vector pP*_CUP1_*-ubc9-CRU [45], which is a low copy yeast - *E. coli* shuttle vector that carries a C_ub_-R-URA3 cassette (CRU), a copper-dependent promoter (P*_CUP1_*) for bait expression, and a HIS3 marker. The human Frizzled 1 coding sequence was amplified by PCR from a human whole brain cDNA preparation (Clontech) using primers Fz1-FW (5′-AATTGTCGACATGGCTGAGGAGGAGGCGCCTAAG-3′) and Fz1-RV (5′-AGCGGCCGCGACTGTAGTCTCCCCTTGTTTGC-3′), introducing *Sal*I and *Not*I restriction sites, respectively. The Fz1 PCR product was eventually used to replace the Ubc9 insert in pP*_CUP1_*-ubc9-CRU using *Sal*I-*Not*I restriction sites, resulting in pP*_CUP1_*-Fz1-CRU.

pP*_CUP1_*-s-Fz1-CRU: The 5′ 138 basepairs of the *Saccharomyces cerevisiae STE2* coding sequence, comprising the receptor's complete N-terminal extracellular tail, were amplified from a yeast genomic DNA preparation using primers Ste2-FW (5′-AATTGTCGACATGTCTGATGCGGCTCCTTC-3′), introducing *Sal*I, and Ste2-RV (5′-GTACTGTTAACTAAACCTTG-3′), introducing a *Hpa*I site. Human Fz1 cDNA lacking the 5′ 333 basepairs of the coding sequence, comprising the region upstream of the receptor's cysteine rich domain (CRD), was amplified from pP*_CUP1_*-Fz1-CRU using primers Fz1-HpaI (5′-ATATGTTAACGACCACGGCTATTGCCAGCC-3′), introducing *Hpa*I, and Fz1-RV. In a triple ligation set up, *Sal*I-*Hpa*I restricted Ste2 fragment and *Hpa*I-*Not*I restricted Fz1 fragment were used to replace the ubc9 insert in pP*_CUP1_*-ubc9-CRU using *Sal*I-*Not*I restriction sites, resulting in pP*_CUP1_*-s-Fz1-CRU.

pYES2-s-Fz1-myc: The s-Fz1 fusion gene was amplified by PCR from pP*_CUP1_*-s-Fz1-CRU using primers DD_11 (5′-ATAAGCTTATGGCTGAGGAGGAGGCGCC-3′) and DD_12 (5′-TAGGATCCCTACAGATCCTCTTCTGAGATGAGTTTTTGTTCGACTGTAGTCTCCCCTTGTTTG-3′), introducing a *Hind*III site, a C-terminal c-myc-tag followed by a STOP codon, and a *BamH*I site, respectively. The *Hind*III-*BamH*I digested s-Fz1-myc fragment was ligated to *Hind*III-*BamH*I restricted yeast expression vector pYES2 (Invitrogen), resulting in pYES2-s-Fz1-myc.

pYES2-s-Fz1-GFP: The s-Fz1 fusion gene was amplified by PCR from pP*_CUP1_*-s-Fz1-CRU using primers DD_13 (5′-ATAAGCTTATGTCTGATGCGGCTCCTTCATTG-3′) and DD_18 (5′-TAGAATTCGACTGTAGTCTCCCCTTGTTTG-3′) introducing *Hind*III and *EcoR*I sites, respectively. Enhanced green fluorescent protein (eGFP) was amplified by PCR from pEGFP-C1 (Clontech) using primers DD_19 (5′-ATGAATTCGTGAGCAAGGGCGAGGAGC-3′) and DD_20 (5′-TAGGATCCTTACTTGTACAGCTCGTCCATGC-3′), introducing *EcoR*I and *BamH*I sites, respectively. *Hind*III-*EcoR*I digested s-Fz1 and *EcoR*I-*BamH*I digested eGFP fragments were ligated to *Hind*III-*BamH*I restricted yeast expression vector pYES2 (Invitrogen), resulting in pYES2-s-Fz1-GFP.

pYES2-s-Fz2-myc: In order to be able to replace the Fz1 *Hpa*I-*EcoR*I fragment from pYES2-s-Fz1-myc with the respective Fz2 fragment, the second *Hpa*I site within the pYES2 vector backbone was first eliminated using GeneTailorTM (Invitrogen) site-directed mutagenesis kit with primers DD_36 (5′-CACTTCAATAGCATATCTTTGTAAACGAAGCATCTG-3′) and DD_37 (5′-AAAGATATGCTATTGAAGTGCAAGATGGAAAC-3′). Human Fz2 cDNA lacking the 5′ 102 basepairs of the coding sequence, comprising the region upstream of the receptor's cysteine rich domain (CRD), was amplified from a Fz2 IMAGE clone (Thomas Schlange, Friedrich Miescher Institute, Basel) using primers DD_38 (5′-TATCGTTAACGACCACGGCTTCTGCCAGCC-3′), introducing *Hpa*I, and DD_39 (5′-GTAGAATTCCTACAGATCCTCTTCTGAGATGAGTTTTTGTTCCACGGTGGTCTCACCGTGTCGGCTGTTG-3′), introducing a C-terminal c-myc tag followed by a stop-codon, and an *EcoR*I site. The *Hpa*I-*EcoR*I - restricted Fz2 fragment was used to replace the Fz1 *Hpa*I-*EcoR*I fragment from pYES2-s-Fz1-myc, resulting in pYES2-s-Fz2-myc.

pYES2-Ste2-Fz1(i), pYES2-Ste2-Fz2(i), pYES2-s-Fz1-Ste2(i) and pYES2-s-Fz2-Ste2(i): Fully synthetic genes for the receptor chimeras between Ste2 and human Frizzled1 or Frizzled2 with sequences as shown in the *Supporting Information* section were ordered at GENEART AG (Regensburg, Germany; Text S1). All sequences were optimized for expression in yeast by adapting to the yeast codon-usage, contained a C-terminal c-myc-tag, and *Hind*III-*EcoR*I sites for subcloning into pYES2.

pYES2-Ste2-Fz1(i)-GFP and pYES2-s-Fz1-Ste2(i)-GFP: The Ste2-Fz1(i) and s-Fz1-Ste2(i) synthetic genes were amplified by PCR using primers DD_13 (5′-ATAAGCTTATGTCTGATGCGGCTCCTTCATTG-3′) and DD_63 (5′-ATGAATTCCAAATCCTCTTCAGAAATCAATTTTTGTTC-3′) introducing *Hind*III and *EcoR*I sites, respectively. Enhanced green fluorescent protein (eGFP) was amplified by PCR from pEGFP-C1 (Clontech) using primers DD_19 (5′-ATGAATTCGTGAGCAAGGGCGAGGAGC-3′) and DD_20 (5′-TAGGATCCTTACTTGTACAGCTCGTCCATGC-3′), introducing *EcoR*I and *BamH*I sites, respectively. *Hind*III-*EcoR*I digested Ste2-Fz1(i) or s-Fz1-Ste2(i) and *EcoR*I-*BamH*I digested eGFP fragments were ligated to *Hind*III-*BamH*I restricted yeast expression vector pYES2, resulting in pYES-Ste2-Fz1(i)-GFP and pYES2-s-Fz1-Ste2(i)-GFP.

pYES2-Ste2-myc: The Ste2 coding sequence was amplified by PCR from a yeast genomic DNA preparation using primers DD_55 (5′-TAAAGCTTAAAAATGTCTGATGCGGCTCCTTC-3′) and DD_56 (5′-GTAGAATTCCTACAGATCCTCTTCTGAGATGAGTTTTTGTTCTAAATTATTATTATCTTCAGTCC-3′), introducing a *Hind*III, and a C-terminal c-myc tag, followed by a STOP codon and a *EcoR*I site, respectively. The *Hind*III-*EcoR*I digested PCR fragment was ligated to *Hind*III-*Eco*RI digested pYES2, resulting in pYES2-Ste2-myc.

pYES2-Edg2-myc: The human Edg2 coding sequence was amplified by PCR from pcDNA3.1TOPOV hEDG2 (S. Marrony, Novartis) using primers DD_9 (5′-ATAAGCTTATGGCTGCCATCTCTACTTC-3′) and DD_10 (5′-TAGGATCCCTACAGATCCTCTTCTGAGATGAGTTTTTGTTCAACCACAGAGTGGTCATTGCTG-3′), introducing a *Hind*III, and a C-terminal c-myc tag, followed by a STOP codon and a *BamH*I site, respectively. The *Hind*III-*Bam*HI digested PCR fragment was ligated to *Hind*III-*BamH*I digested pYES2, resulting in pYES2-Edg2-myc.

### Yeast methodologies

Standard yeast methodologies were used as described in [46]. For yeast transformations the LiAc/carrier DNA/PEG method was employed [47].

### Expression growth test for Fz1- and s-Fz1 - Ura3p fusion proteins

Yeast strain JD53 [38] was transformed with pP*_CUP1_*-Fz1-CRU or pYES2-s-Fz1-myc, and plated equally onto SD-HIS, and SD-HIS-URA minimal media plates containing Cu_2_SO_4_ (100 µM) as inducer of protein expression.

### Growth modulation assays upon receptor overexpression on solid medium

Yeast strain MH272-1da or MH272-1da *far1::hphMX4* were transformed each with equal amounts of plasmid DNA of receptor expression constructs as indicated in the results section, and plated onto SD-URA minimal medium agar plates. Resulting single clones were inoculated into liquid SD-URA minimal medium, and incubated over night. Yeast cultures were spotted in serial dilutions (∼1E4, 1E3, 1E2 cells per spot) onto SD-URA (glucose containing) and SD+galactose-URA agar plates, and incubated as indicated in the results section.

### Growth modulation assays upon receptor overexpression in liquid medium, and budding index determinations

Yeast strain MH272-1da transformed with plasmid DNA of receptor expression constructs as indicated in the results section was grown in SD-URA medium (glucose containing). Growth of exponentially growing cultures was monitored using optical density determination (OD 600 nm) as measured in a BIOTEK Synergy HT plate reader. Aliquots of the exponentially growing cultures in SD-URA were pelleted using centrifugation and washed twice with, and finally resuspended in, SD+galactose-URA medium. The budding index of each culture was calculated as ratio of number of budded cells to total cells as determined using a hemocytometer slide at the indicated timepoints over 32 hours.

### Mating pathway activation growth assay on solid medium

Yeast strains MH272-1da, MH272-1da *far1::hphMX4* and MH272-1da *ste2::hphMX4* were incubated over night in SD complete minimal medium (SDC), and were spotted in serial dilutions (∼1E4, 1E3, 1E2 cells per spot) onto SDC agar plates in duplicates. 1 µg α-factor (Sigma) was used to treat one dilution series of each strain by spotting directly onto the yeast patches.

### Mating pathway activation P*_FUS1_*-luciferase assays

The P*_FUS1_*-firefly luciferase reporter system was described previously [21], and was used here with the following modifications for two different assay modes. For both modes, the newly devised yeast strain MC18 *ste2::hphMX4 sst2::kanMX4* was employed. The strain was sequentially co-transformed with pRHF-luc (carrying the P*_FUS1_*-luciferase reporter module), pRGP-Gpa1 (for Gpa1 expression under its native promoter from a low copy plasmid; [19]) or the respective empty parent plasmid pRS413 [48], and the pYES2-based receptor expression constructs as indicated in the results section. Plating was done onto repressive SD-TRP-URA-HIS minimal medium.

One assay mode was particularly devised for strain-to-strain comparisons of the basal P*_FUS1_*-luciferase levels: Single clones were inoculated into 10 mL SD-TRP-URA-HIS medium and incubated over night. Subsequently, the culture was divided 1∶1, cells were pelleted, washed twice with 10 mL sterile water, resuspended in 5 mL SD-TRP-URA-HIS medium, or inducing 5 mL SD+galactose-TRP-URA-HIS medium, and incubated for 6 or 24 hours at 30°C (as indicated in the results section). Cells were pelleted again and washed with 10 mL water. The pellet was eventually resuspended in 300 µL phosphate buffer saline (PBS) supplemented with a protease inhibitor cocktail (1 complete miniTM protease inhibitory tablet from Roche per 50 mL PBS). Acid-washed glass beads (0.45 µm, Sigma) corresponding to a volume of ∼200 µL were added, and cells were disrupted using a swinging mill (Qiagen) at maximal amplitude for 5 minutes. The resulting crude protein extract was cleared by centrifugation at 3.000×g for 5 minutes at 4°C. 10 µL of the extract were assayed in a total volume of 100 µL PBS (including protease inhibitor cocktail) in duplicates for luciferase activity using a BIOTEK Synergy HT device to inject 100 µL of luciferase assay reagent (Promega) in 96-well format. The luciferase levels were normalized to total protein content as determined using the Biorad protein assay.

The second assay mode was used for the ligand concentration-response experiments, and represents a simplified assay procedure for increased throughput. Single clones were inoculated into 50 mL SD-TRP-URA-HIS medium and incubated to exponential growth phase. Subsequently, cells were pelleted, washed twice with 50 mL sterile water, and resuspended in inducing 50 mL SD+galactose-TRP-URA-HIS, and incubated for 4 hours at 30°C to allow for receptor expression. Subsequently, the optical density (OD600nm) of the culture was adjusted to 0.2–0.4 as measured in a BIOTEK Synergy HT plate reader. 500 µL aliquots of the culture were prepared in a 24-well cell culture plate (TPP, Switzerland), and treated with α-factor (Sigma), recombinant carrier-free mouse Wnt-3a or Wnt-5a (R&D Systems) as indicated in the results section for 4 hours at 30°C. For a removal of the medium, the aliquots were transferred to 2 mL Spin-X® centrifuge tube cellulose acetate filters (Corning Costar), and centrifuged at 3.000×g for 1 minute. The cells were resuspended in 500 µL deionized water, and 100 µL aliquots were arrayed in quadruplets in a transparent flat-bottom 96-well plate (Nunc). OD-measurement of the plate was performed optionally to control proper pipetting. From this step on, all subsequent pipetting steps were performed using a multi-channel pipette. The cell wall was digested for 30 minutes at 37°C by adding 50 µL of zymolyase 100T (SEIKAGAKU) stock solution in water (40 mg mL^−1^) supplemented with a protease inhibitor cocktail (1 complete miniTM protease inhibitory tablet from Roche per 50 mL solution). Eventually, 50 µL tissue-culture luciferase-assay lysis buffer (Sigma) was used to disintegrate the cells by repeated up-and-down pipetting. Finally, 50 µL aliquots were assayed for luciferase-activity as described above.

### Western blot analysis

Yeast strain MH272-1da [41] transformed with constructs as indicated in the result section was cultivated in 50 mL SD-URA medium to the exponential growth phase. To induce protein expression, the cultures were transferred to galactose-containing full medium (YPGal) for 6 hours. Cells were pelleted, and resuspended in 300 µL lysis buffer (100 mM Tris-HCl pH 8.0, 20% glycerol, 1% Triton X-100, 1 mM dithiothreitol, 1 mM phenylmethylsulphonylfluoride). Cells were disrupted by vortexing at 4°C for 10 minutes following addition of acid-washed glass beads (0.45 mm diameter). The crude protein extract was cleared by centrifugation at 3000×g for 5 minutes. The protein concentration was determined using the Biorad protein assay. 20 µg extract were electrophoresed using a 4–12% gradient NuPAGE Bis-Tris gel system (Invitrogen). The proteins were electroblotted to a nitrocellulose membrane. Anti-c-myc detection using a monoclonal antibody from Roche (clone 9E10) was performed either through enhanced chemiluminescence (Biorad Immun-Star HRP substrate kit; and Hyperfilm from Amersham Biosciences) or chromogenic detection (Invitrogen Western Breeze kit). As loading control, a rabbit anti-Kss1p polyclonal antibody (Santa Cruz) was used at 1∶200 dilution, and detected using the Invitrogen Western Breeze kit.

### Confocal microscopy

Microscopic inspections of agarose-embedded galactose-induced s-Fz1-GFP, Ste2-Fz1(i)-GFP or s-Fz1-Ste2(i)-GFP expressing yeast strain MH272-1da were performed using a Zeiss LSM510 confocal microscope.

## Supporting Information

Text S1 (0.03 MB DOC)
