# Supplementary material for: Signaling of Human Frizzled Receptors to the Mating Pathway in Yeast
Source: PLoS One. 2007 Sep 26;2(9):e954. doi: 10.1371/journal.pone.0000954 (PMC1978518; doi:10.1371/journal.pone.0000954)
Supplement: Text S1 — (0.03 MB DOC) [file pone.0000954.s001.doc]

**Text S1:**

Sequences of the synthetic genes as ordered at GENEART (Regensburg, Germany):

Ste2-Fz1(i)

aagcttaaaaatgtctgatgctgctccatctttgtctaatttgttttatgatccaacttataatcctggtcaatctactattaactatacttctatctatggtaatggttctactattacttttgatgaattgcaaggtttagttaattctacagttactcaagctataatgtttggtgttagatgtggtgctgctgctttgactttgattgttatgtggatgacttctagaagtagaagtaatcaagtttctttgtttttgattattttgcattctgctttgtattttaagtatttgttgtctaattattcttctgttacttatgctttgactggttttccacaattcatttctagaggtgatgttcatgtttatggtgcaactaacattattcaagttttgttggttgcttctattgaaacttctttggtttttcaaattaaatggggtcatgaagcatttaagagaattggtttaatgttgacttctatttcttttactttgggtattgctacagttacaatgtattttgtttctgctgttaaaggaatgattgttacttataatgatgtttctgctactcaagataagtactttaatgcttctacaattttgttagcttcttctattaattttatgtcttttgttttggttgttaaattgattttggctattagatctagaagatttggttttgtttctttgtttagaattagaactattatgaaacatgatggtactggtttaaaacaatttgattcttttcatattttgttgattatgtcttgtcaatctttgttggttccatctattattttcattttggcttattctttaaaaccaaatcaaggtacagatgttttgactacagttgctactttgttggctgttttgtctttgccattatcttctatgtgggctactgctgcttcttggagaaaattctatactagattgactaactcaaaacaaggtgaaactactgttgaacaaaaattgatttctgaagaggatttgtaagaattc

Ste2-Fz2(i)

aagcttaaaaatgtctgatgctgctccatctttgtctaatttgttttatgatccaacttataatcctggtcaatctactattaactatacttctatctatggtaatggttctactattacttttgatgaattgcaaggtttagttaattctacagttactcaagctatcatgtttggtgttagatgtggtgctgctgctttgactttgattgttatgtggatgactagtagatcaaggaggaatcaagtttctttgtttttgattattttgcattctgctttgtatttcaagtatttgttgtctaattattcttctgttacttatgctttgactggttttccacaatttatttctagaggtgatgttcatgtttatggtgctactaatattattcaagttttgttggttgcttctattgaaacttctttggtttttcaaattaaatggggtcatgaagcatttaagagaattggtttaatgttgacttctatttcttttactttgggtattgctacagttacaatgtattttgtttctgctgttaaaggaatgattgttacttataatgatgtttctgctactcaagacaaatactttaatgcttctacaattttgttagcttcttctattaattttatgtcttttgttttggttgttaaattgattttggctattagaagtagaagatttggttttgtttctttgtttagaattagaactattatgaaacatgatggtactggtttaaaacaatttgattcttttcatattttgttgattatgtcttgtcaatctttgttggttccatctattattttcattttggcttattctttaaaaccaaatcaaggtacagatgttttgactacagttgctactttgttggctgttttgtctttgccattatcttctatgtgggctactgctgcttcttggagaaaattctatactagattgactaattctagacatggtgaaactactgttgaacaaaaattgatttctgaagaggatttgtaagaattc

s-Fz1-Ste2(i)

aagcttaaaaatgtctgatgctgctccatctttgtctaatttgttttatgatccaacttataatcctggtcaatctactattaactatacttcaatttatggtaatggttctactattacttttgatgaattgcaaggtttagttaatgatcatggttattgtcaaccaatttctattccattgtgtactgatattgcttataatcaaactattatgccaaatttgttgggtcatactaatcaggaagatgctggtttagaagttcatcaattttatccattggttaaagttcaatgttctgctgaattaaagtttttcttgtgttcaatgtatgctccagtttgtactgttttggaacaagcattgccaccatgtagatctttgtgtgaaagagctagacaaggttgtgaagcattgatgaacaaatttggttttcaatggccagatactttgaaatgtgaaaaatttccagttcatggtgctggtgaattgtgtgttggtcaaaatacttctgataaaggtactccaacaccatctttgttgccagaattttggacttctaatccacaacatggtggtggtggtcatagaggaggttttccaggtggtgctggtgcttctgaaagaggaaaattttcttgtccaagagctttgaaagttccatcttatttgaattatcattttttgggtgaaaaagattgtggtgctccatgtgaaccaactaaagtttatggtttaatgtattttggtccagaggaattgagattttctagaacttggattggtatttggtctgttttgtgttgtgcttctactttgtttactgttttgacttatttggttgatatgagaagatttaaaactccaattttcattatctatccagaaagaccaattatatttttgtctggttgttatactgctgttgctgttgcttatattgctggttttttgttggaagatagagttgtttgtaatgataaatttgctgaagatggtgctagaacagttgctcaaggtactaaaaaggaaggttgtactattttgtttatgatgttgtatttcttttctatggcttcttctatttggtgggttattttgtctttgacttggtttttggctgctggaatgaaagttatttttacaggtgataatattgaagctaattctcaatattttcatttggctgcttgggctgttccagctattaaaactattactattttggctttgggtcaagttgatggtgatgttttgtctggtgtttgttttgttggattgaacaatgttgatgctttgagaggttttgttttggctccattgtttgtttatttgtttattggtacttcttttttgttggctttaaaaacagaaaaattggaaaaattaatggttagaattggtgttttcagtgttttgtatacagttccagctactattgttattgcttgttatttttatgaacaagcatttagagatcaatgggaaagatcttgggttgctcaatcttgtaaatcttatgctattccatgtccacatttgcaagctggtggtggtgctccaccacatccaccaatgtctccagattttacagtttttatgatcaagtacttaatgactttgattgttggtattacttctggtttttggatttggtctggtaaaactttgaacaacaatgcttctaaaactaatacaattacttctgattttactacttctactgatagattttatccaggaactttatcttcttttcaaactgattctattaacaatgatgctaaatcttctttgagatctagattatatgatttgtatcctagaaggaaggaaactacatctgataaacattctgagagaacttttgtttctgaaactgctgatgatattgaaaaaaatcaattttatcaattaccaactccaacttcttctaaaaatactagaattggtccttttgctgatgcttcttataaggaaggtgaagttgaaccagttgacatgtatactccagatactgctgctgatgaagaggctagaaaattttggacagaagataacaacaatttggaacaaaaattgatttctgaagaggatttgtaagaattc

s-Fz2-Ste2(i)

aagcttaaaaatgtctgatgctgctccatctttgtctaatttgttttatgatccaacttataatcctggtcaatctactattaactatacttcaatttatggtaatggttctactattacttttgatgaattgcaaggtttagttaatgatcatggtttttgtcaaccaatttctattccattgtgtactgatattgcttataatcaaactattatgccaaatttgttgggtcatactaatcaagaggatgctggtttagaagttcatcaattctatccattggttaaagttcaatgttctccagaattgagattctttttgtgttcaatgtatgctccagtttgtactgttttggaacaagctattccaccatgtagatctatttgtgaaagagctagacaaggttgtgaagcattgatgaacaagtttggttttcaatggccagaaagattgagatgtgaacattttccaagacatggtgctgaacaaatttgtgttggtcaaaatcattctgaagatggtgctccagctttgttgactactgctccaccaccaggtttacaaccaggtgctggtggtactccaggtggtccaggtggtggaggagcacctccaagatatgctactttggaacatccatttcattgtccaagagttttgaaagttccatcttatttgtcttataagtttttgggtgaaagagattgtgctgctccatgtgaaccagctagaccagatggttcaatgtttttttctcaagaggaaactagatttgctagattgtggattttgacttggtctgttttgtgttgtgcttctactttttttactgttactacttatttggttgatatgcaaagatttaaaactccaattttcattatctatccagaaagaccaattatctttttgtctggttgttatactatggtttctgttgcttatattgctggttttgttttgcaagagagagttgtttgtaacgaaagattttctgaagatggttatagaactgttgttcaaggtactaaaaaagagggttgtactattttgtttatgatgttgtattttttttctatggcttcttctatttggtgggttattttgtctttgacttggtttttggctgctggaatgaaagttatttttacaggtgataatattgaagctaattctcaatattttcatttggctgcttgggctgttccagctgttaaaactattactattttggctatgggtcaaattgatggtgatttattatctggtgtttgcttcgttggtttaaattctttggatccattgagaggttttgttttagctccattgtttgtttatttgtttattggtacttcttttttgttagctttaaaaacagaaaaattggaaagattaatggttagaattggtgtttttagtgttttgtatactgttccagctactattgttattgcttgttatttttatgaacaagcatttagagaacattgggaaagatcttgggtttctcaacattgtaaatctttggctattccatgtccagctcattatactcctagaatgtctccagattttactgtttacatgattaagtatttgatgactttgattgttggtattacttctggtttttggatttggtctggtaaaactttgcataacaatgcttctaaaactaatacaattacttctgattttactacttctactgatagattttatcctggtactttatcttcttttcaaactgattctattaacaatgatgctaaatcttctttgagatctagattatatgatttgtatcctagaaggaaggaaactacttctgataaacattctgagagaacttttgtttctgaaactgctgatgatattgaaaaaaatcaattttatcaattaccaactccaacttcttctaaaaatactagaattggtccttttgctgatgcttcttataaggaaggtgaagttgaaccagttgacatgtatactccagatactgctgctgatgaagaggctagaaaattttggacagaagataacaacaatttggaacaaaaattgatttctgaagaggatttgtaagaattc

Amino acid sequences of the receptor chimeras between yeast Ste2 and human Frizzled 1 or Frizzled 2 receptors. Highlighted are the predicted transmembrane segments (underlined), the intracellular portions of Fz1, Fz2 or Ste2 (bold), as well as the Ste2p signal sequence (italics). All four receptor chimeras contain a C-terminal c-myc tag:

Ste2-Fz1(i)

msdaapslsnlfydptynpgqstinytsiygngstitfdelqglvnstvtqaimfgvrcgaaaltlivmwmtsrsr**s**nqvslfliilhsalyfkyllsnyssvtyaltgfpqfisrgdvhvygatniiqvllvasietslvfqik**wghea**fkriglmltsisftlgiatvtmyfvsavkgmivtyndvsatqdkyfnastillassinfmsfvlvvklilairsrrf**gfvslfrirtimkhdgt**glkqfdsfhillimscqsllvpsiifilayslkpnqgtdvlttvatllavlslplssmwataa**swrkfytrltnskqgettv**eqkliseedl

Ste2-Fz2(i)

msdaapslsnlfydptynpgqstinytsiygngstitfdelqglvnstvtqaimfgvrcgaaaltlivmwmtsrsr**r**nqvslfliilhsalyfkyllsnyssvtyaltgfpqfisrgdvhvygatniiqvllvasietslvfqik**wghea**fkriglmltsisftlgiatvtmyfvsavkgmivtyndvsatqdkyfnastillassinfmsfvlvvklilairsrrf**gfvslfrirtimkhdgt**glkqfdsfhillimscqsllvpsiifilayslkpnqgtdvlttvatllavlslplssmwataa**swrkfytrltnsrhgettv**eqkliseedl

s-Fz1-Ste2(i)

*msdaapslsnlfydptynpgqstinytsiygngstitfde*lqglvndhgycqpisiplctdiaynqtimpnllghtnqedaglevhqfyplvkvqcsaelkfflcsmyapvctvleqalppcrslcerarqgcealmnkfgfqwpdtlkcekfpvhgagelcvgqntsdkgtptpsllpefwtsnpqhgggghrggfpggagasergkfscpralkvpsylnyhflgekdcgapceptkvyglmyfgpeelrfsrtwigiwsvlccastlftvltylvdmrrf**ktpifii**yperpiiflsgcytavavayiagflledrvvcndkfaedgartvaqgtkkegctilfmmlyffsmassiwwvilsltwflaagmk**viftgdn**ieansqyfhlaawavpaiktitilalgqvdgdvlsgvcfvglnnvdalrgfvlaplfvylfigtsflla**l**ktekleklmvrigvfsvlytvpativiacyfyeqafrdqwerswvaqscksyaipcphlqagggapphppmspdftvfmikylmtlivgitsgfwiwsgktln**nnasktntitsdfttstdrfypgtlssfqtdsinndaksslrsrlydlyprrkettsdkhsertfvsetaddieknqfyqlptptsskntrigpfadasykegevepvdmytpdtaadeearkfwtednnnl**eqkliseedl

s-Fz2-Ste2(i)

*msdaapslsnlfydptynpgqstinytsiygngstitfde*lqglvndhgfcqpisiplctdiaynqtimpnllghtnqedaglevhqfyplvkvqcspelrfflcsmyapvctvleqaippcrsicerarqgcealmnkfgfqwperlrcehfprhgaeqicvgqnhsedgapallttapppglqpgaggtpggpggggappryatlehpfhcprvlkvpsylsykflgerdcaapceparpdgsmffsqeetrfarlwiltwsvlccastfftvttylvdmqrf**ktpifii**yperpiiflsgcytmvsvayiagfvlqervvcnerfsedgyrtvvqgtkkegctilfmmlyffsmassiwwvilsltwflaagmk**viftgdn**ieansqyfhlaawavpavktitilamgqidgdllsgvcfvglnsldplrgfvlaplfvylfigtsflla**l**kteklerlmvrigvfsvlytvpativiacyfyeqafrehwerswvsqhckslaipcpahytprmspdftvymikylmtlivgitsgfwiwsgktlh**nnasktntitsdfttstdrfypgtlssfqtdsinndaksslrsrlydlyprrkettsdkhsertfvsetaddieknqfyqlptptsskntrigpfadasykegevepvdmytpdtaadeearkfwtednnnl**eqkliseedl
